# New therapeutic aspects of steroidal cardiac glycosides: the anticancer properties of Huachansu and its main active constituent Bufalin

**DOI:** 10.1186/s12935-019-0806-1

**Published:** 2019-04-11

**Authors:** Chien-shan Cheng, Jiaqiang Wang, Jie Chen, Kuei Ting Kuo, Jian Tang, Huifeng Gao, Lianyu Chen, Zhen Chen, Zhiqiang Meng

**Affiliations:** 1 Department of Integrative Oncology, Fudan University Shanghai Cancer Center, Shanghai, 200032 China; 2 Department of Oncology, Shanghai Medical College, Fudan University, Shanghai, 200032 China; 3 School of Chinese Medicine, LKS Faculty of Medicine, The University of Hong Kong, Pokfulam, Hong Kong SAR China; 4 Faculty of Anesthesiology, Changhai Hospital, Naval Medical University, Shanghai, 200433 China; 5 Department of Anaesthesiology, Fudan University Shanghai Cancer Center, Shanghai, 200032 China; 6 Department of Orthopaedics, Shanghai Institute of Traumatology and Orthopaedics, Ruijin Hospital, Shanghai Jiaotong University School of Medicine, Shanghai, 200025 China

**Keywords:** Bufalin, Huachansu, Anti-cancer, Traditional Chinese Medicine

## Abstract

**Aim of the review:**

In the past decade, increasing research attention investigated the novel therapeutic potential of steroidal cardiac glycosides in cancer treatment. Huachansu and its main active constituent Bufalin have been studied in vitro, in vivo and clinical studies. This review aims to summarize the multi-target and multi-pathway pharmacological effects of Bufalin and Huachansu in the last decade, with the aim of providing a more comprehensive view and highlighting the recently discovered molecular mechanisms.

**Results:**

Huachansu and its major derivative, Bufalin, had been found to possess anti-cancer effects in a variety of cancer cell lines both in vitro and in vivo. The underlying anti-cancer molecular mechanisms mainly involved anti-proliferation, apoptosis induction, anti-metastasis, anti-angiogenesis, epithelial–mesenchymal transition inhibition, anti-inflammation, Na^+^/K^+^-ATPase activity targeting, the steroid receptor coactivator family inhibitions, etc. Moreover, the potential side-effects and toxicities of the toad extract, Huachansu, and Bufalin, including hematological, gastrointestinal, mucocutaneous and cardiovascular adverse reactions, were reported in animal studies and clinic trails.

**Conclusions:**

Further research is needed to elucidate the potential drug–drug interactions and multi-target interaction of Bufalin and Huachansu. Large-scale clinical trials are warranted to translate the knowledge of the anticancer actions of Bufalin and Huachansu into clinical applications as effective and safe treatment options for cancer patients in the future.

## Introduction

Traditional Chinese Medicine (TCM) is a medical practice with more than 2500 years of history in China. It has recently been recognized as a new type of chemotherapy adjuvant that can improve the efficacy of chemotherapy and ameliorate the side effects of cancer chemotherapies. Ever since the launch of chemotherapy for tumor treatment, natural products have become an important source of the development of novel cancer therapies. Although there is no pathological concept of cancer under TCM theory, it is easy to find the descriptions of cancer-like symptoms in the ancient medical documents described as lumps, bumps, and toxins, termed “Chuang, Yong, Zhong and Du (疮痈肿毒)” in Chinese [1]. Therefore, identifying drugs used to treat lumps and bumps, as well as other cancer-related symptoms, such as relief from fever, diarrhea, vomiting, and pain, may link the traditional use of the agent to the modern pathological concept of cancer and may strengthen the pharmacological relevance of TCM to contemporary anti-cancer treatments.

Chansu (CS, Senso in Japanese) is the dried secretion from the skin glands of *Bufo bufo gargarizans* Cantor or *Bufo melanostictus* Schneider [2]. According to the principles of TCM theory, CS is commonly used to counteract toxicity, alleviate pain, and induce resuscitation [3, 4]. It can be considered as an anti-infectious agent for pyogenic infection induced unconsciousness and may be related to its anti-inflammatory and anti-microbial effects [3, 5]. In TCM practice, CS is prescribed to patients with “heat and toxins” syndrome, which refers to the modern concepts including acute gastroenteritis, severe vomiting, diarrhea, abdominal pain, high fever, carbuncles, lumps, and bumps [6]. Huachansu (HCS) is an injectable form of the sterilized hot-water extract of CS [7]. It is manufactured by Anhui Jinchan Biochemistry Company Ltd., in Huaibei, China [Chinese Food and Drug Administration, FDA (ISO9002)] and is widely used for inflammatory diseases as well as for the treatment for various types of cancer, including liver, lung, pancreatic, and colorectal cancers in China [8–12].

The molecular basis for the anti-inflammatory effect of HCS is proposed to be the bioactive steroidal cardiac glycosides [13]. Indeed, glycosides isolated from HCS have been shown to possess blood pressure stimulation, respiratory excitation, anti-inflammatory, anesthetic, and anti-neoplastic activities [14]. HCS and its derived single compounds may achieve their anti-inflammatory effects by modulating nuclear factor-κB (NF-κB) signaling and down-regulating inflammatory-related genes such as cyclooxygenases, lipoxygenases, inducible nitric oxide synthase, and thereby decrease nitric oxide and prostaglandin E_2_ (PGE_2_) production [9, 11–13]. In cancerous cells, glycosides derived from HCS also exhibit cytostatic and cytotoxic activities, induce cellular apoptosis, inhibits angiogenesis, reverses chemotherapeutic drug resistance, and modulate immune responses. Previous studies suggest that Na^+^/K^+^ pump or sodium- and potassium-activated adenosine 5′-triphosphatase (Na^+^, K^+^-ATPase) is a potential drug target that contributes to the selective control role of cardiac glycosides in tumor proliferation, but does not affect normal cell growth [10, 15, 16]. Moreover, accumulating evidence reveals the anti-cancer effect of HCS and its derived single compounds in several tumor types in vitro and in vivo.

**Fig. 1:**
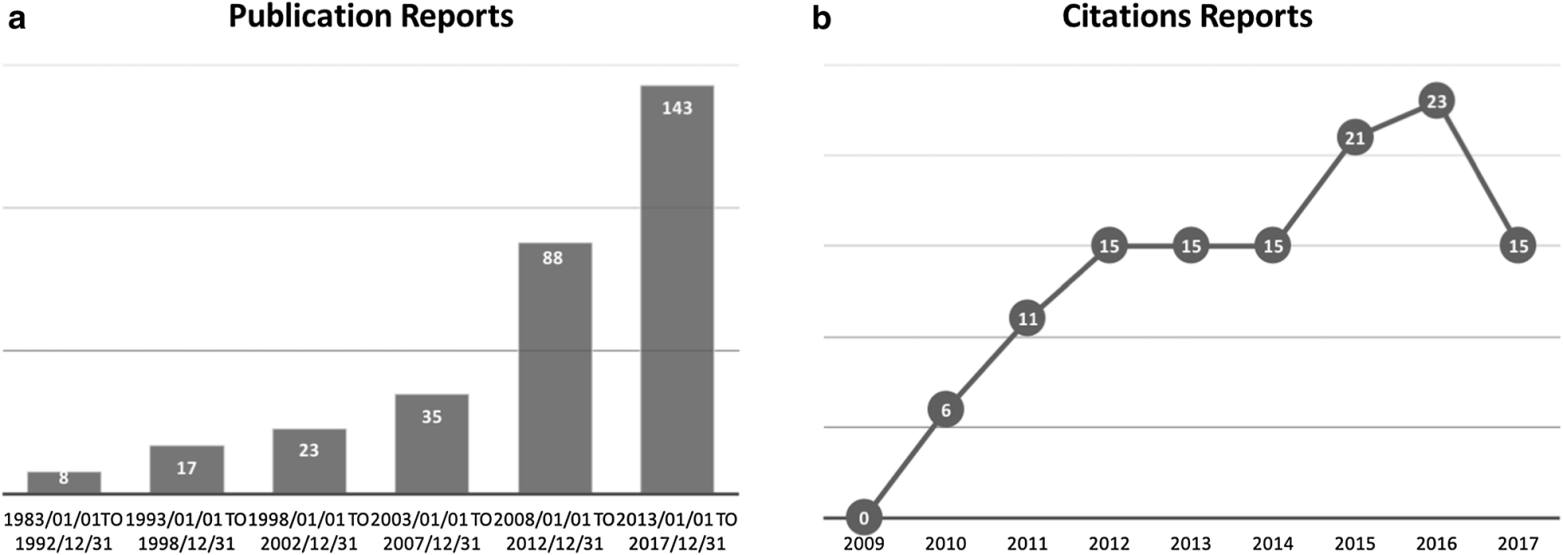
**a** Increase of publication in number in the recent 10 years; Data was retrieved from PubMed in 5-year interval with keywords of (“Neoplasms”[MeSH] AND “bufalin” OR “huachansu” OR “chansu” OR “chan-su”). **b** Steady annual citation of our phase II clinical trial since its publication in recent 5 years

Furthermore, in the last decade, some studies have proposed new properties and effects of HCS, Chansu and their major active constitutes, bufalin, in the treatment of cancer (Fig. 1). Interestingly, there are an increasing number of studies investigating both in vitro and in vivo experiments in the recent 5 years, indicating an increased awareness of the translational potential of HCS and its derived steroidal cardiac glycosides in animal studies. In this review article, data on the anti-cancer effect of HCS and its major active constitutes bufalin published in the recent 10-years were retrieved from databases including PubMed, MEDLINE, CNKI, and clinicaltrial.gov. This review focuses on the anti-cancer pharmacological effects and mechanisms of action of HCS and bufalin, with emphasis on elaborating the translational potential and future clinical application. This review article also discusses the recent studies on drug delivery and its derivatives.

## The bioactive constituents of Huachansu

The chemical composition and pharmacological activity of HSC have been investigated since the 1980s [7, 14, 15, 17, 18]. HSC contains two primary bioactive chemical components, indole alkaloids (bufotenine, bufotenidine, cinobufotenine, and serotonin), and steroidal cardiac glycosides [7, 14, 15, 18]. Their extraction rate is mainly determined by the extraction method. High performance liquid chromatography (HPLC) quantitative analysis confirmed that the aqueous extract of HSC yield around 20-fold higher serotonin than bufadienolides (75.7 ± 0.1 mg/g and 3.8 ± 0.0 mg/g, respectively), while methanol or ethanol extraction solution contains 5–26 times higher concentrations of bufadienolides, with only trace amounts of serotonin [14]. So far, there are more than 28 steroidal cardiac glycosides identified from HCS [19]. The investigation into the potential use of cardiac glycosides in cancer therapeutic was initiated more than 40 years ago, yet was abandoned due to the toxicities [20]. However, in 1999, Scandinavian oncologist Haux [21] reported that digoxin induced tumor cell apoptosis in a variety of human cancer cell lines at non-toxic concentration. Recent studies demonstrated that steroidal cardiac glycosides are the major anti-neoplasm component of HCS.

**Fig. 2:**
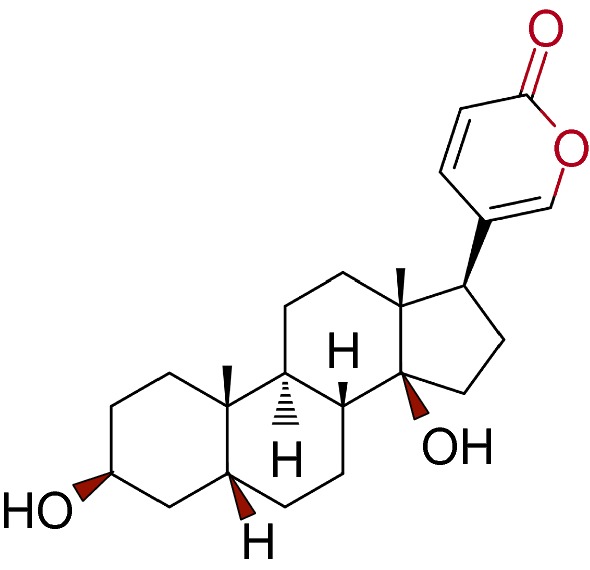
Shows the structure of Bufalin (C_24_H_34_O_4_, Pubchem CID: 9547215). Retrieved from National Center for Biotechnology Information. PubChem Compound Database; CID = 9547215, from https://pubchem.ncbi.nlm.nih.gov/compound/9547215 (accessed 22 Aug 2018)

Bufalin (PubChem CID: 9547215, Chemical formula C_24_H_34_O_4_, Fig. 2) is a cardiac glycoside and the major active component is attributed to the anti-tumor activity of HCS [12]. Similar to other bufadienolides (such as resibufogenin, cinobufagin, and bufotalin), Bufalin is a cardioactive C-24 steroid characterized by an α-pyrone ring at C-17 [22, 23]. Bufalin exhibits a variety of biological activities. Its structural similarity with digitoxin accounts for both the therapeutic effect as well as the unwanted side-effects such as cardiotonic, blood pressure stimulatory and respiratory stimulatory effects in cancer treatment [20, 24, 25]. Recent studies emerge on purified compounds and cardiac glycosides. Bufalin may represent a promising form of targeted cancer chemotherapy for long-term applications without severe side effects.

## Cytostatic effects of Huachansu and Bufalin on tumors

It appears that the cytostatic effect of Bufalin and HCS is well demonstrated in a variety of tumors such as breast, colorectal, gastric, lung, liver and bone tumors. The 50% inhibition dose (IC50) among cancer cell lines after 1–3 days of treatment are summarized in Table 1. The in vitro effects of Bufalin and HCS in arresting cell cycle, inducing intrinsic and extrinsic apoptosis are reproducible across studies. Recent studies have confirmed that Bufalin can lead to cell cycle redistribution, at least in part by its role in the inhibition of Na^+^/K^+^-ATPase activity, in various types of human cancers [26, 27]. In cancer cells, Bufalin can arrest cell cycle at G0/G1 and G2/M phase, owing to the various dosage of treatment and cell lines [28–30]. The sensitivity to Bufalin treatment varies among cell lines. Currently, there is a lack of in-depth study to predict the exact effect of Bufalin on cell cycle. Moreover, it is worth noting that one article published in 2013 by Clifford and Kaplan suggested that Bufalin and other cardiotonic steroids, including Ouabain and Digitoxin, can inhibit membrane Na^+^/K^+^-ATPase activity in multiple cell lines regardless of metastatic potential [31, 32]. Also, human breast tumor cells are more resistant to growth inhibition and apoptosis induction of cardiotonic steroids than non-tumorigenic breast cells [31]. The 50% inhibitory dose with 24-h of Bufalin treatment in metastatic invasive ductal carcinoma MCF10CA1 cell and the invasive estrogen receptor (ER)-negative MDA-MD-231 breast cancer cell is two- and threefold, respectively, the dosage of human noncancerous mammary epithelial cells 184D and 184A1 [31]. Another study on the effect of HCS on normal human lymphocytes was marginal (with more than 60% viability at 48-h) compared with A-549, Jurkat, MCF-7 cancer cells (with less than 10% viability) at a dose of 0.16 mg/mL of CS extract [33]. However, a majority of studies investigating the cytostatic effect of HCS and Bufalin are carried out solely on tumorigenic cells instead of normal non-cancerous cells. Further investigation is warranted to elucidate the pharmacological action of Bufalin treatment.

**Table 1:** Growth inhibition of Bufalin (BF) on human cancer cell lines after 24–72H treatment

Cancer type	Cell line	24H IC50	48H IC50	72H IC50	Refs.
Breast	MCF-7	317.9 ± 1.5 nM	46.5 ± 1.4 nM		Yan et al. [125]
		3.2 nM			Wang et al. [56]
	MCF-7/ADR		100.17 ± 30.16 nmol/L		Wang et al. [126]
	MCF-10A	465.2 ± 25.9 nM			Clifford et al. [31]
	MCF10CA1	635.2 ± 171.8 nM			Clifford et al. [31]
	MDA-MB-231	934.1 ± 2.0 nM	513.3 ± 1.6 nM		Yan et al. [125]
			263.3 ± 68.24 nmol/L		Wang et al. [126]
		936.4 ± 4.9 nM			Clifford et al. [31]
				20.0 nM	Song et al. [55]
	MDA-MB-231-LM3-3			16.6 nM	Song et al. [55]
	SUM149PT			16.6 nM	Song et al. [55]
	SUM159PT			15.9 nM	Song et al. [55]
Cervical	Hela	154 ± 21.5 nM	37.5 ± 2.15 nM	11.8 ± 2.13 nM	Pan et al. [127]
Colorectal	SW620	76.72 ± 6.21 nmol/L	34.05 ± 4.21 nmol/L	16.7 ± 6.37 nmol/L	Zhu et al. [128]
		287.35 ± 4.34 nM	57.63 ± 7.80 nM	20.39 ± 1.95 nM	Zhang et al. [129]
	HCT116	82.6 μmol/L			Qiu et al. [130]
		0.243 μM	0.024 μM		Wang et al. [131]
	LoVo	56.778 ± 7.34 nM	11.48 ± 2.89 nM	6.64 ± 2.79 nM	Zhang et al. [129]
Endometrial	Ishikawa		0.7 ng/mL		Takai et al. [132]
	HHUA		0.5 ng/mL		Takai et al. [132]
	HEC-1B		0.6 ng/mL		Takai et al. [132]
Esophageal	Eca-109			1.0 μM	Lv et al. [133]
	EC9706			1.0 μM	Lv et al. [133]
	TE5			2.6 μM	Lv et al. [133]
	TE11			4.9 μM	Lv et al. [133]
	Hec2			3.8 μM	Lv et al. [133]
Gallbladder	GBC-SD		48.12 ± 2.03 nM	28.23 ± 1.78 nM	Jiang et al. [45]
	SGC996		125.03 ± 5.16 nM	102.78 ± 3.21 nM	Jiang et al. [45]
Gastric	SGC7901	> 800 nmol/L			Li et al. [134]
	MGC803	160 ± 0.87 nmol/L			Li et al. [134]
Liver	HepG2	182.30 ± 13.78 nM			Li et al. [27]
		307 ± 87 nM			Ozdemir et al. [84]
			33.65 nmol/L		Gao et al. [60]
			143.2 nM		Miao et al. [49]
	PLC/PRF/5	52.20 ± 14.16 nM			Li et al. [27]
			157.87 nmol/L		Gao et al. [60]
	SMMC7721	97.74 ± 8.83 nM			Li et al. [27]
	SK-HEP-1	110.33 ± 5.32 nM			Tsai et al. [135]
Leukemia	NB4	40 nmol/L	27 nmol/L	17 nmol/L	Zhai et al. [136]
	K562			0.0943 μmol/L	Zhai et al. [136]
	K562/VCR			0.0401 μmol/L	Zhai et al. [136]
	HEL		0.046 μmol/L		Wang et al. [137]
Lung	A549	4.5 nM			Wang et al. [56]
			56.14 ± 6.72 nmol/L	15.57 ± 4.28 nmol/L	Zhu et al. [138]
				8.15 ± 0.69 nmol/L	Sun et al. [92]
		22.00 ± 3.53 nM	10.20 ± 1.01 nM		Liu et al. [36]
Melanoma	A375.S2		450.38 nM		Hsiao et al. [44]
Oral	CAL27	125 nM			Tsai et al. [139]
		122.6 nM			Tian et al. [140]
Osteosarcoma	U-2OS	0.297 μM			Zhang et al. [141]
			8.49 ± 2.1 μg/L		Yin et al. [142]
	U-2OS/MTX300		10.19 ± 1.7 μg/L		Yin et al. [142]
	Saos-2	0.318 μΜ			Zhang et al. [141]
Ovarian	SK-OV-3		1.0 ng/mL		Takai et al. [132]
	OMC-3		0.6 ng/mL		Takai et al. [132]
Tongue	SCC-4		300 nM		Chou et al. [143]
Pancreatic	Capan-2	159.2 nM			Tian et al. [140]
Non-cancerous	Breast epithelial cell 184D	384.6 ± 36.5 nM			Clifford et al. [31]
	Breast epithelial cell 184A	295.9 ± 10.5 nM			Clifford et al. [31]
	Mouse normal hepatocyte			No cytotoxicity at 10 μM	Song et al. [55]
	HUVECs	53.1 nmol/L			Qiu et al. [130]
	Het-1A^a^			10.1 μM	Lv et al. [133]
	PBMCs^b^		16.5 ± 4.7 ng/mL		Yuan et al. [144]
	Primary normal human endometrial epithelial cells		Little sensitivity to BF from 0.1 to 10 ng/mL		Takai et al. [132]
	PBMCs		16.5 ± 4.7 ng/mL		Yuan et al. [144]

^a^Human normal esophageal squamous cells

^b^Normal peripheral blood mononuclear cells (PBMCs) were isolated from three healthy volunteers (32 ± 9 years of age)

## In vivo evaluation of Huachansu and its derivative Bufalin on tumor inhibition

To evaluate the anticancer potential of Bufalin in vivo, various xenograft mice models are carried out throughout cancer types such as breast, cervical, colorectal, liver, gallbladder, lung, and pancreatic cancer. The results are very compelling that Bufalin can inhibit xenograft tumor growth, increase sensitivity to chemotherapy, and prolong the survival rate of mice. Most of the studies reported a dosage of 0.1–2 mg/kg intraperitoneally (i.p.) administered at a frequency of once per day 5 days per week to every 3 days for a period ranging from 12 days to 6 weeks in mice xenograft model without inducing significant weight loss or adverse effects, as summarized in Table 2. In the recent 10 years, although in vivo studies have been increasingly used to assess the effect of Bufalin. Most of the studies have used the xenografts models and reported the attenuation of tumor growth rate as well as the reduction of tumor weight. Yet, the lack of orthotropic models in the Bufalin research led to a relative shortage in the evaluation of the systemic effect of Bufalin contributing to not only the tumor itself but to the microenvironment or tumor metastasis. Further studies are warranted to elucidate the related effects.

**Table 2:** In vivo evaluation of Bufalin in mouse tumor models

Cancer type	Drugs	Animal	Tumor models	Transplantation	Treatment	Results	Refs.	Year
Bone	Bufalin	Athymic nude mice; male; 7–8 weeks old	Rat breast sarcocarcinoma Walker 256 cell	Inoculate 2 × 10^5^ cell into the intramedullary space of the mouse femur	1 day after tumor inoculation, given by i.p. 0.5, 1, 1.5, or 2 mg/kg/day for 21 days	Cancer-induced pain relief; 50% reduction in bone tissue injury	Ji et al. [83]	2017
Cervical	Bufalin	BALB/c nude mice; female; 4–5 weeks old	Human cervical squamous cell carcinoma Siha cell	Subcutaneously inoculate 3 × 10^6^ cell	After reaching an average tumor volume of 100 mm^3^, given by i.p. Bufalin 10 mg/kg ± paclitaxel (10 mg/kg per 4 days) for 32 days	Bufalin synergizes with paclitaxel to inhibit tumor growth without apparent loss of body weight	Liu et al. [80]	2016
Breast	Bufalin-NP	SCID mice; female; 6–7 weeks old	Human breast MDA-MB-231-LM3.3 cell	Inject 0.75 × 10^6^ cell into one of the second mammary fat pads	6 days after tumor injections, given by i.v. Bufalin-NP (1.5 mg/kg) three times per week for 20 days	Bufalin sensitizes cancer cells to MK-2206 and blocks tumor growth	Wang et al. [56]	2014
	Bufalin-BCS-NP	Balb/c nude mice; female; 4 weeks old	Human breast MCF-7 cell	Subcutaneously implant 1 × 10^7^ cell into the right axilla skin	7–10 days after tumor inoculation, (1) given by i.p. BF-BCS-NPs (1 mg/kg BF equivalent in PBS) or free BF (1 mg/kg in ethanol) every 2 days for 20 days; (2) give by i.v. BF-BCS-NPs (5 mg/kg BF equivalent in PBS) at D1, D3	Free BF suppresses tumor growth accompanying with a significantly decreased body weight. BF-BCS-NPs suppresses tumor growth without apparent loss of body weight	Tian et al. [104]	2014
	3-Phospho-bufalin	Nude mice; female; 4–6 weeks old	Human breast MDA-MB-231-LM3-3 cell	Inject 0.75 × 10^6^ cell into one of the second mammary fat pads	14 days after injection, given by s.c. phospho-BF (0.75 mg/kg per dose, 3 doses per week) for 3 weeks	2.4 times reduction in tumor weight; no cardiotoxicity observed	Song et al. [55]	2015
	Bufalin	Athymic nude mice; female; 5 weeks old	Human breast MDA-MB-231 cell	Subcutaneously inject 5 × 10^6^ into both dorsal flank regions	4 weeks after injection, (1) given by intra-tumoral injection BF 10 μL (1 mM, in 0.9% normal saline) to the left flank for 4 weeks; (2) 10 μL volume of normal saline to the right flank for 4 weeks	Significantly enhances breast cancer xenograft growth; promote the inflammatory response	Chen et al. [38]	2017
Colorectal	Bufalin-loaded mPEG-PLGA-PLL-cRGD nanoparticles (BNPs)	Athymic nude mice; female; 4–6 weeks old	Human colon SW620 cell	Inject 5 × 10^6^ cell into the dorsal subcutaneous space	Bufalin-loaded mPEG-PLGA-PLL-cRGD nanoparticles (BNPs) containing 1 mg/kg bufalin, given by injection through the vena caudalis once a day for 14 days	Suppresses tumor growth; bufalin-loaded NPs significantly enhanced treatment efficacy compared to that of a bufalin water solution	Yin et al. [105]	2012
	Bufalin	Male athymic nude mice	HCT116-luc-vector and HCT116-luc-miR-497	Inject 1 × 10^6^ cells intravenously via the tail vein	1 week after injection, given by Bufalin for 1 mg/kg via the tail vein (three times a week) for 5 weeks	Inhibited colorectal cancer metastasis; improved life of survival. Improved physiological characteristics in terms of body weight, skin roughness, mental status, and survival rate	Qiu et al. [130]	2014
	Bufalin-loaded pluronic polyetherimide nanoparticles	Male athymic nude mice	HCT116	Injecte 1 × 10^6^ cells intravenously via the tail vein.	2 weeks after injection, (1) given by of Bufalin 1 mg/kg; (2) given by 20 mg/kg of Bufalin-loaded pluronic PEI nanoparticles via the tail vein (0.2 mL per mouse, three times per week) for 3 weeks	Inhibited colorectal cancer metastasis. Improved quality of life and physiological characteristics in terms of body weight, skin roughness, and mental status	Hu et al. [93]	2014
	Bufalin	BALB/c mice; male; 5–6 weeks old	HCT116	Inject 2 × 10^10^ cells s.c. into the right axillary region. Tumor mode of the second generation: 2 weeks after injection, harvest the s.c. xenogra tumors, cut into pieces (1.5 mm in diameter), implant into the axillary region s.c. Orthotopic xenogra model: harvest third generation s.c. tumors and cut into pieces (1.5 mm in diameter)	NS group (treated with 0.2 mL normal saline); 5-Fu group (treated with 5-FU, 25 mg/kg); low Bufalin group (0.5 mg/kg); medium Bufalin group (1.0 mg/kg); high Bufalin group (1.5 mg/kg). NS, 5-FU, and Bufalin were administrated by intraperitoneal injection, once per day from day 15 to day 21 (12 mice in each group)	Inhibit cell growth. Lower tumor volume. Prolong survival time	Wang et al. [131]	2015
	Bufalin-DOX	Athymic nude mice (BALB/c-nu/nu) of 6–8 weeks	HCT8/ADR	Inject 1 × 10^7^ cells s.c. under the shoulder in the nude mice	Mice were randomized into six groups (6 in each group) when the tumor volumes reached 150–200 mm^3^: control; BF (0.1 mg/kg, i.p., q3d × 5); DOX (0.1 mg/kg, i.p., q3d × 5); DOX (0.5 mg/kg, i.p., q3d × 5); DOX (1.0 mg/kg, i.p., q3d × 5); DOX (0.1 mg/kg, i.p., q3d × 5) plus BF (0.1 mg/kg, i.p., q3d × 5, given 1 h before DOX administration)	BF remarkable increased the effect of DOX against the ABCB1 resistant HCT8/ADR colorectal cell xenografts in nude mice	Yuan et al. [145]	2015
	Bufalin	Male nude mice (BALB/c nu/nu, 5-week-old)	HCT116	Inject 2 × 10^6^ cells into the subcutaneous tissues.	2 weeks after injection, (1) given by cisplatin (10 mg/kg body weight) i.p. every 3 days for 4 weeks.; (2) given by bufalin (1 mg/kg body weight) i.p. every 3 days for 4 weeks	Inhibition of tumor growth and tumor tissue weights are greater with the combination of cisplatin and bufalin than with cisplatin alone. Tumors treated with the combination of cisplatin and bufalin showed more cell vacuolization and nuclear shrinkage than with cisplatin alone	Sun et al. [146]	2017
Gallbladder	Bufalin	Male athymic nude mice (5 week-old)	GBC-SD	Xenograft model: Inoculate 1 × 10^6^ GBC-SD cells into the left axillary region	24 h postinoculation, given by PBS i.p. and bufalin with (0.1, 0.2, and 0.4 mg/kg) i.p. every 2 days for up to 20 days	Suppression of tumor growth	Jiang et al. [45]	2014
Liver	Bufalin	BALB/c nu/nu mice (18–20 g, 5 week-old)	HCCLM3	Inject 5 × 10^6^ cells s.c. into the upper left flank region of nude mice. Revmove the tumors when reached approximately 1 cm in length (approximately 4 weeks after injection) and mince into small pieces of equal volume (1.5–2 × 2 × 2 mm^3^), then transplant into the livers of 24 nude mice	From day 8 to 38, given by1 mg/kg Bufalin; 1.5 mg/kg Bufalin; 100 mg/kg LY294002 and saline i.p. thrice weekly, respectively	1.5 mg/kg Bufalin decreased the sizes and qualities of orthotopic transplanted tumors as well as pulmonary metastasis. Orthotopic transplanted tumor tissues were necrotic and the apoptotic cell number was markedly higher in 1.5 mg/kg Bufalin group. Inhibition of AKT/GSK3β/β-catenin/E-cadherin signaling pathways	Zhang et al. [72]	2014
	Bufalin–sorafenib	BALBc nu/nu mice (6 week-old)	Human HCC cell lines SMMC7721	Inoculate 5 × 10^6^ cells s.c. into the abdominal intraderma	Control group: inject with the vehicle i.p. (5 days/week, 2 weeks). Experimental group: (1) injection of 1 mg/kg bufalin i.p. (5 days/week, 2 weeks); (2) oral uptake of 30 mg/kg/day sorafenib (5 days/week, 2 weeks); (3) the combination of both injections of bufalin i.p. and oral uptake of sorafenib (5 days/week, 2 weeks)	Inhibit blood vessel formation in the intradermal tumors, manifested by the vessel numbers and branches and attenuate tumor weight in nude mice with the combination treatment	Wang et al. [64]	2016
	Bufalin	BALBc nu/nu mice (6 week-old)	Human HCC cell lines SMMC7721	Inoculate 5 × 10^6^ cells s.c. into the right flank	Tumor size was measured every 4 days after the treatment. Tumor-bearing mice were sacrificed after 16 days of treatment, and the tumor weight was evaluated	Combination treatment inhibits tumor growth and tumor angiogenesis in vivo. The combination treatment group showed more reduced microvessel density than any other group	Wang et al. [64]	2016
	Bufalin	BALBc nu/nu mice (6 week-old)	SMMC7721-GFP	Inoculate 5 × 10^6^ cells s.c. into the right flanks. 4 weeks later, cut the non-necrotic tumor tissue into 1 mm^3^ pieces and orthotopically implanted into the liver. In addition, inject 2 × 10^6^ via mouse tail veins	(1) Inject 1 mg/kg bufalin i.p. (5 days/week for six weeks); (2) Inject PBS i.p. (5 days/week for 6 weeks)	Bufalin reduced liver/lung metastases. Bufalin inhibited invasion through EMT	Wang et al. [76]	2016
Myeloma	Bufalin-MK2206	BALB-c nu/nu female mice (4–6 week-old); NOD-SCID female mice (4–6 week-old)	MOPC315; H929	Inject 2 × 10^7^ MOPC315 cells s.c. in the right flanks of the BALB-c nu/nu mice. Inject 1 × 10^7^ cells H929 cells s.c. in the right hind leg of NOD/SCID mice	Mice bearing MOPC315 MM tumors were treated with bufalin (1 mg/kg; intraperitoneally) daily in the presence and/or absence of MK2206 (120 mg/kg orally) for 10 days. Mice injected with H929 MM cells were treated with 1 mg/kg bufalin daily with or without 120 mg/kg MK2206 for 12 days	Bufalin combined with MK2206 blocked MM tumor growth, decreased tumor cell proliferation and increased the percentage of apoptotic cells	Xiang et al. [147]	2017
Osteosarcoma	Bufalin	BALB/c nude mice; female; 6 week-old	U2OS/MTX300	Inject 5.6 × 10^6^ cells s.c. into the axilla of the mice	10 days after injection, (1) control group: 100 mL of vehicle i.p.; MTX group: MTX (250 mg/kg) with calcium leucovorin rescue (24 mg/kg at 16, 20, or 24 h after MTX) i.p. per week; (3) low Bufalin group: 0.75 mg/kg i.p.; (4) High Bufalin group:1.5 mg/kg i.p.	Bufalin inhibited tumor growth	Xie et al. [86]	2012
Pancreas	Bufalin	BALB/c nu/nu mice; male; 4-week-old	Mia PaCa-2	Inject 6 × 10^6^ cells s.c. into the back of mice	When tumors reached the size of 100 mm^3^: (1) vehicle alone (control); (2) Bufalin (0.1 mg/kg, for 10 days); (3) Gemcitabine (125 mg/kg, 3 times/week for 2 weeks); (4) Bufalin and Gemcitabine in combination	Bufalin potentiates the anti-tumor effect of gemcitabine in vivo. Combination treatment with gemcitabine signicantly reduced the tumor volume and cell proliferation activity	Chen et al. [148]	2012
	Bufalin	BALBc nu/nu mice; female; 6-week-old	MiaPaCa2/GEM	Inoculated 2 × 10^5^ cells into the right flanks of mice	Given by (1) injections of 1.5 mg/kg bufalin (5 days/week) for 4 weeks; (2) injections with vehicle (20 μL saline) for 4 weeks. In addition, MiaPaCa2/GEM cells (2 × 10^6^) was given to one of another two groups pre-treated with bufalin via the tail veins for 6 weeks	Inhibit pancreatic tumor growth	Wang et al. [82]	2016
	Bufalin	BALBc nude mice; male; 5-week-old)	BxPC3-luc2	Injected 1 × 10^7^ cells s.c. into the left buttock of mice	7 days after inoculation, control group: inject saline i.p.; Bufalin groups: inject 1 mg/kg and 2 mg/kg for 14 days; Positive control: DDP 2 mg/kg every other day i.p. for 14 days	Bufalin treatment inhibits tumor growth	Liu et al. [149]	2016
Lung	Bufalin	BALB/c nu/nu mice; male; 6–8 week-old	NCI-H460	Inject 1.3 × 10^7^ cells s.c. into flank of each mouse	When tumor volume exceeded 100 mm^3^, given by (1) vehicle (0.1% DMSO); (2) Bufalin: 0.1, 0.2, or 0.4 mg/kg for 14 days	Bufalin suppresses tumor growth	Wu et al. [150]	2017

*NP* nanoparticles, *BSC* biotin modified chitosan, *s.c.* subcutaneously, *i.p.* intraperitoneally

The acute toxicity measured as 50% lethal dose values for Bufalin in mice when administered intraperitoneally was approximately 2.2 mg/kg, as first reported in1960 by Okada et al. [34] by Wang et al. in 2003 [35] and by Liu et al. in 2016 [36], consistently. Bufalin showed the lowest toxicity when injected intraperitoneally among principle active components, including cinobufagin and resibufogenin, isolated from CS [34]. When administered intravenously (i.v.) to rabbits, Bufalin induced an elevation of blood pressure accompanied by marked respiratory excitation. Yet, although Bufalin is structurally slimier to digitoxin that both of them possess the same aglycone except for the lactone ring in 17-position, digitoxin does not induce respiratory excitation induced by the i.v. administration of Bufalin was not observed with digitoxin intervention at a dosage as high as 0.2 mg/kg via i.v. injection [34]. A study in 1995 examined the toxicity and teratogenicity of CS using a single i.p. injection into maternal mice during the embryonic organogenesis [37]. At doses below 50 mg (dry weight)/kg body weight, no detectable changes were observed in both maternal mice and fetuses; however, at a dose of 50 mg/kg, adverse effects were found with a reduction in maternal mice body weight, liver, and kidney structural abnormalities, and an increased number of resorbed and dead fetuses [37].

It is worth noting that most studies have reported a significant reduction in tumor growth rate and tumor weight after i.p. injection of Bufalin. An article reported a contradictory result in a breast cancer xenograft model with an intra-tumoral injection of Bufalin in 2017 [38]. The results showed that intra-tumoral injection of 10 μL of 1 μM Bufalin, three times a week for 4 weeks, equivalent to 3.87 ng per mice, significantly enhanced tumor growth by promoting inflammatory response compared with intratumoral injection of saline via increasing expression of COX-2/IL-8, promoting p-65 NF-κB translocation and modulating mitogen-activated protein kinase (MAPK) pathways [38]. This contradictory result may be due to the difference in not only the route of administration but also the difference in the equivalent dose among studies.

As previously reported, for example, Han, et al. conducted a study in 2007 and reported an anti-tumor effect of Bufalin with i.p. injection of 0.5, 1.0 or 1.5 mg/kg/day for 10 days in mice hepatocellular carcinoma (HCC) xenograft model with a body weight around 18–20 g, which is equivalent to an i.p. injection of 10 to 30 mg/mice of Bufalin throughout the course of treatment [39]. Other studies, as summarized in Table 2, reported anti-tumor effect with i.p. administration of doses ranging from 0.5 to 1.5 mg/kg at a frequency of daily injection for 10 days, every other day injection for 20 days or once every 3 days for 4 weeks. There appears to be more than a 1000-fold difference between the anti-tumor effect reported by i.p. injection and the pro-inflammatory or tumor-promoting effects upon intra-tumoral injection. A variation in doses is also found. According to the material and safety data sheet, the median lethal dose of Bufalin administered to mice by i.v. injection was 0.74 mg/kg [38]. It is plausible that the contradictory results on the tumor suppressing or the tumor-promoting effects of Bufalin may be due to the variation among different cancer cell lines, different route of administration, or different doses used. In future research, more in-depth study using the same treatment protocol is needed to better assess the role of Bufalin in cancer treatment.

## Molecular mechanisms of anti-tumor activity

The cell cycle arresting, intrinsic (also known as mitochondria-mediated) and extrinsic (also known as receptor-mediated) apoptosis-inducing effects of Bufalin at concentrations ranging from 1 nM to 10 μΜ, such as intracellular reactive oxygen species (ROS) production, caspase-dependent apoptosis, modulating MAPK signaling cascade, inhibiting NF-κB signaling, are well not only well established among various cancer cell lines [29, 40–53]. The steroid receptor coactivator (SRC) family, including SRC-1, SRC-2, and SRC-3, is involved in molecular and physiological processes across diseases by activating nuclear receptors and other transcriptional factors, such as NF-κB. SRCs are frequently overexpressed in malignancies and are associated with cancer cell proliferation, invasion, and metastasis [54–56]. Previous studies suggested that Bufalin promotes SRC-3 protein degradation in breast, gastric and lung cancer [36, 55–58]. Moreover, Bufalin suppressed triple negative breast cancer proliferation at a nanomolar concentration (~ 3–5 nM), which the concentration of which digoxin required to inhibit SRC-3/SRC-1 is greater than 200 nM [56]. The effective concentration of Bufalin to inhibit SRCs is within the on cardiac toxicity concentration reported in patients plasma (~ 9 nM) [12]. Furthermore, the water soluble prodrug of Bufalin, 3-phospho-bufalin, inhibits the growth of orthotopic triple negative breast cancer [55]. All in all, Bufalin is a potent and safe steroid receptor coactivators inhibitor.

**Fig. 3:**
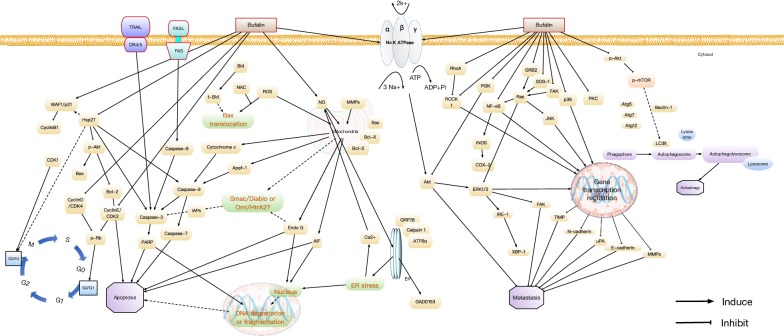
A schematic summary of the molecular mechanisms of Bufalin-mediated anti-cancer effect

Although the exact mechanism of the anti-cancer effect of Βufalin has hitherto been unknown. It is worth noting that several novel mechanisms contributing to the anti-cancer effects of Bufalin are proposed and increasingly studied in the recent 10 years, including sensitization of TRAIL-mediated apoptosis, autophagic cell death induction, reversing chemotherapy drug resistance, suppressing cellular invasion, migration, and anti-adhesion, inhibiting epithelial–mesenchymal transition, as shown in Table 3. A schematic summary of the molecular mechanism of Bufalin-mediated anti-cancer effect is shown in Fig. 3.

**Table 3:** Summary on the pharmacological action of Huachansu extract (HCS) and Bufalin (BF) reported in recent 10 years

Cancer type	Study type	Pharmacological action	Pathways involved	Refs.	Year
Bladder	In vitro	Growth inhibition (50–300 nM, 24H); G2/M arrest; apoptosis induction (T24, EJ) [BF]	Bax/Bcl-2 ratio Fas, DR4, DR5, TRAIL, PARP cleavage↑; pro-caspase-3, -8, -9, Bcl-xL, Bid, XIAP, cIAP-1, cIAP-2↓	Hong et al. [43]	2012
	In vitro	Growth inhibition (50–200 nM, 24H); G0/G1 arrest; mitochondrial/apoptosis (T24) [BF]	ROS production, cytochrome c, Apaf-1, AIF, caspase-3, -7, -9, Bax↑; ΔΨm, cyclin D, CDK4, cyclin E, CDK2, phospho-Rb, phospho-AKT Bcl-2↓	Huang et al. [29]	2012
	In vitro	Migration and invasion inhibition (5–100 nM, 24H) (T24) [BF]	TIMP-1, -2, phospho-ERK↑; claudin-2, -3, -4, MMP-2, -9, Active-MMP-2, -9↓	Hong et al. [151]	2013
	In vitro	Sensitization of TRAIL-mediated apoptosis (2.5–10 nM, 24H) (T24) [BF]	Caspase-9, pro-caspase-9, DR5, degradation of poly (ADP-ribose) polymerase↑	Kang et al. [152]	2017
Bone	In vivo	Relieves cancer-induced pain and bone destruction (Walker 256 cells) [BF]	OPG↑; RANKL, serum TRACP5b, ICTP, PINP↓	Ji et al. [83]	2017
Breast	In vitro	Sensitization of TRAIL-mediated apoptosis (0.02–0.25 μM, 24H) (MCF-7, MDA-MB-231) [BF]	Mcl-1, Bcl-xL, p-STAT3↓	Dong et al. [153]	2011
	In vitro	Growth inhibition (50 nM, 24H); apoptosis induction; enhanced TRAIL-induced apoptosis (MCF-7, MDA-MB-231) [BF]	DR4, DR5, p-ERK, p-JNK, p-p38, cleaved-PARP↑; pro-caspase-8, Cbl-b↓	Yan et al. [125]	2012
	In vitro and in vivo	Growth inhibition (1–5 nM, 24H); promote coactivators’ protein degradation (MCF-7) [BF]	SRC-3 mRNA expression, SRC-3 protein degradation↑; intrinsic transcriptional activities of SRC-1 and SRC-3↓	Wang et al. [56]	2014
	In vitro	Sensitization of TRAIL-mediated apoptosis (50 nmol/L, 24H); redistributing death receptors in lipid rafts (MCF-7, MDA-MB-231) [BF]	DR4, DR5, cleaved-PARP, cleaved-caspase-8↑; caspase-8↓	Yan et al. [154]	2014
	In vitro and in vivo	Growth inhibition (100 ng/mL, 8H); mitochondrial/apoptosis (MCF-7) [BF, BF-BCS-NPs]	Intracellular ROS, BAX, cleaved-caspase-3↑; ΔΨm, Bcl-2, survivin↓	Tian et al. [104]	2014
	In vitro	Growth inhibition; apoptosis (10–50 nmol/L, 48H) (MCF-7/ADR, MDA-MB-231)[BF]	PARP cleavage, miR-155-5p expression↑; DNMT1, DNMT3a, FOXO3a↓	Wang et al. [126]	2016
	In vitro and in vivo	Growth inhibition (12–200 nM) (LM3-3) [BF, phospho-BFs]	Synergizes with Gefitinib; SRC-3↓	Song et al. [55]	2015
	In vitro	Growth inhibition (4–8 nM, 48H) (MDA-MB-231); enhanced HDAC inhibitors induced apoptosis [BF]	↑;SRC-3p-Akt, Bcl-2↓	Zou et al. [155]	2016
	In vitro and in vivo	Promote inflammatory response (0.001–1 μM, 12H) (MDA-MB-231) [BF]	p65 translocation; PKC-induced COX-2 and IL-8, PGE2, p-JNK, p-p38, p-ERK, TPA-induced MMP-3 protein and mRNA expression↑	Chen et al. [38]	2017
Cervical	In vitro and in vivo	Growth inhibition (0.05–0.2 μM, 24H); G2/M arrest; apoptosis; migration and invasion inhibition (0.01–0.04 μM, 24H) (Siha, Hela) [BF]	BAX, P21, p27, E-cadherin, GSK3β↑;Bcl-2, Bcl-xL, cyclin A, cyclin B1, CDK2, MMP-9, SNAIL1, integrin α2, integrin β5, FAK, p-FAK(Tyr397), p-GSK3β(Ser389), AKT1, p-AKT(Ser473)↓	Liu et al. [80]	2016
	In vitro	Growth inhibition (0–50 nM, 24H); apoptosis induction (Hela) [BF]	HSP27, vimentin, HNRPK↓	Pan et al. [127]	2012
Colorectal	In vitro	Growth inhibition (25–100 nM, 48H); G2/M arrest; autophagy induction (HT-29, Caco-2) [BF]	LC3-II, ROS, Atg-5, Beclin-1, p-JNK2↑	Xie et al. [59]	2011
	In vitro	Growth inhibition (20, 80 nmol/L, 24H); G2/M arrest; apoptosis (SW620) [BF]	PARP cleavage, cleaved-caspase-3, BAX/BCL-2 ratio↑;p-Stat3, p-ERK, livin↓	Zhu et al. [128]	2012
	In vitro	Growth inhibition (100 nM, 9H); mitotic arrest; G2/M arrest (HT-29, HCT-116) [BF]	p-H3↑;HIF-1α, NF-κB, Plk1 expression↓	Xie et al. [156]	2013
	In vitro and in vivo	Anti-migration and anti-metastasis (3.12–50 nM, 12H) (HCT116), ex vivo micro vessel sprouting (HUVECs) [BF]	miR-497 expression↑; VEGFA expression↓	Qiu et al. [130]	2014
	In vitro and in vivo	Growth inhibition (0.03–3 μΜ, 24–48H); G2/M arrest; apoptosis (HT116) [BF]	PTEN, Bad, caspase-3 phosphorylation, caspase-3 cleavage↑; p-PTEN, p-AKT↓	Wang et al. [131]	2015
	In vitro	Anti-proliferation and anti-migration (10–50 nM, 48H); induce apoptosis; G2/M arrest (LoVo, SW620) [BF]	Cyclin B1, p–cdc2, p21, cleaved-PARP, Bax, cleaved-caspase-7, -9, E-cadherin↑; Bcl-2, N-cadherin, β-catenin, CPSF4, hTERT↓; inhibit hTERT by down-regulating CPSF4	Zhang et al. [129]	2016
	In vitro and in vivo	Suppress growth of cisplatin-resistant cell rather than sensitive-one; reverse ABCB1-mediated multi-drug resistance (5–20 nM, 48H) (LoVo/ADR, HCT8/ADR, HCT8/ABCB1) [BF]	ATPase activity of ABCB1↑; ABCB1↓	Yuan et al. [145]	2015
	In vitro and in vivo	Reverse cisplatin drug resistance (5 nM, 48H) (HCT116, LoVo HCT116-STSCscis, LoVo-STSCscis) [BF]	CD133, CD44, OCT4, SOX2, NANOG, ABCG2 ↓	Sun et al. [146]	2017
Gallbladder	In vitro	Apoptosis induction (25–200 nmol/L, 48H); S-phase arrest; mitochondrial dysfunction induction (GBC-SD, SGC996) [BF]	Cleaved-caspase-3, -9, cleaved-PARP ↑; Cyclin A, Cyclin B1, Cyclin D1, CDK1, NF-κB, Bcl-2, ΔΨm↓	Jiang et al. [45]	2014
Gastric	In vitro	Growth inhibition (100, 200 nmol/L, 24H); G2/M phase arrest; intrinsic apoptosis induction (SGC7901, MGC803) [BF]	SPARC antagonizes bufalin-induced apoptosis; p-Src, p-Akt, p-ERK↑; ΔΨm, cyclin B1, cyclin A↓	Li et al. [134]	2015
	In vitro	Growth inhibition (25–200 nmol/L, 48H); apoptosis induction (SGC7901, MGC803)[BF]	Bax, cleaved-PARP↑; Bcl-2, pro-caspase-3, miR-298 suppressed apoptosis↓	Zhao et al. [157]	2015
	In vitro	Proliferation inhibition (100 nM, 48H); cisplatin-sensitization; apoptosis induction (SGC7901, MKN-45, BGC823) [BF]	p-AKT, p-GSK3β, p-mTOR, p-4EBP1, p-S6 K↓	Zhao et al. [158]	2016
	In vitro	G0/G1 phase arrest (50, 80 nmol/L, 48Η); caspase and mitochondrial-mediated apoptosis induction; ER stress induction; protective autophagy activation (SGC7901, BGC823) [BF]	Cleaved-caspase-3, cleaved-PARP, Bax/Bcl-2, CHOP, p-eIF2a, p-JNK, LC3-II, Atg5, Beclin-1↑; LC3-I, p62↓	Zhao et al. [159]	2017
Glioma	In vitro	Growth inhibition (20–80 nM, 24H); mitochondria-mediated apoptosis induction; autophagy activation (U87MG, LN229) [BF]	ROS production, Bax, cytosolic cytochrome *c*, cleaved-PARP, cleaved-caspase-3, -4, LC3-II, p-AMPK, p-ACC, ATF6f, p-PERK, p-IRE1α, p-eIF2α, GRP78, GRP994, CHOP↑; Bcl-2, p-mTOR, p-4EBP1, p-p70S6K, PERK↓	Shen et al. [160]	2014
	In vitro	Proliferation inhibition (5–40 μM, 24H); cancer stem cell-like phenotypes inhibition; apoptosis induction (U251, U87) [BF]	miR-203↑;OCT4, SOX2, SPARC↓	Liu et al. [161]	2017
HCC	In vitro	Growth inhibition (0.1–1 μΜ, 2H) (HepG2, PLC/PRF/5, SMMC7721) [BF]	p-ERK, p-Akt, ATP1A3↑; FoxO3a↓	Li et al. [27]	2011
	In vitro	Growth inhibition (0.001–0.1 μmol/L, 24–72H); induction of fas- and mitochondria-mediated apoptosis (HepG2) [BF]	Bax, cytochrome *c*, cleaved-caspase-3, -9, PARP cleavage ↑; ΔΨm, Bcl-2, pro-caspase-3, -9, -10, Bid ↓	Qi et al. [162]	2011
	In vitro	Anti-proliferation (50–250 nM, 48Η); enhances the anti-cancer effects of Sorafenib (6.25lM) (PLC/PRF/5, HepG2) [BF]	Enhanced apoptotic cell death in combination with Sorafenib; p-Akt↑;p-ERK↓	Gao et al. [60]	2012
	In vitro	Growth inhibition (50–100 nM, 24H); G2/M arrest; autophagy induction (SK-HEP-1)[CS]	Chk1, Wee1, LC3-II, Atg5, Atg7, Atg12, Beclin-1↑; Cyclin A, cyclin B, CDK1, p-CDK1(Thr161), Cdc25c, p-CDC25c(Ser198), p-Akt(Ser308), p-AKT(Ser473), p-mTOR(Ser2481), AKT kinase activity↓	Tsai et al. [135]	2012
	In vitro	Growth inhibition (10–100 nM, 48H); autophagy induction; apoptosis induction (HepG2) [BF]	p-APMK, Beclin-1, LC3-II, p-p70S6K↑; p-mTOR, p62↓	Miao et al. [49]	2013
	In vitro	Growth inhibition; G2/M arrest (0.04 μM, 4–12H); autophagy induction (Huh7, Hep3B, HA22T) [BF]	TNF, BECN-1, MAPK, ATG8↑; Bcl-2, Bid↓	Hsu et al. [30]	2013
	In vitro	Growth inhibition; anti-migration (10, 100 nmol/L, 48H); anti-invasion; anti-adhesion (HCCLM3, HepG2) [BF]	GSK3β, E-cadherin↑; p-Akt, p-GSK3β, MMP-9, -2, β-catenin nuclear translocation↓	Qiu et al. [71]	2013
	In vitro	Growth inhibition (5, 10 nM, 48H); anti-migration and invasion (SK-Hep1) [BF]	PI3K, p-Akt, NF-κB translocation, MMP-2, -9, FAK, Rho A, VEGF, MEKK3, MKK7, uPA↓	Chen et al. [68]	2013
	In vitro	Growth inhibition (50–100 nmol/L, 48H); apoptosis induction; ER stress induction; autophagy induction (Huh-7, HepG2) [BF]	Beclin-1, p-JNK1, p-JNK2, IRE1, ATG5, LC3-I, LC3-II↑; p62↓	Hu et al. [163]	2014
	In vitro	Reverse multidrug resistance (1 nM, 48H); G0/G1 phase arrest (BEL-7402/5-FU) [BF]	drug efflux pump activity, TS, MRP1, Bcl-xL/Bax ratio ↓	Gu et al. [164]	2014
	In vivo	Orthotopic growth inhibition, anti-metastasis (1 mg/kg and 1.5 mg/kg) (HCCLM3-R) [BF]	p-Akt, GSK3β, E-cadherin↑; p-GSK3β, β-catenin, MMP-9, -2↓	Zhang et al. [72]	2014
	In vitro	Reverse Sorafenib resistance (50–200 nM, 48H); synergies with sorafenib to induce apoptosis (HepG2, HepG2-Sora, Huh7, Huh7-Sora) [BF]	IRE1, CHOP, P-eIF2α↑; p-Akt↓	Zhai et al. [165]	2015
	In vitro	Anti-invasion and metastasis (0.085 μg/mL, 72H) (BEL-7402) [BF]	E-cadherin, ALB↑; β-catenin, p-GSK-3β Ser9, MMP-7, COX-2, Cyclin D1, AFP↓	Gai et al. [66]	2015
	In vitro and in vivo	Synergies anti-angiogenic effect of sorafenib (2.5–10 nM, 48H); anti-migration; S-phase arrest (HUVECs, SMMC7721) [BF]	p-ERK↑; VEGF, p-Akt, p-mTOR↓	Wang et al. [64]	2016
	In vitro and in vivo	Inhibits TGF-β1 induced EMT and invasion (10 nM, 24–72H) (SMMC7721) [BF]	E-cadherin↑; N-cadherin, Vimentin, Snail, HIF-1α↓	Wang et al. [76]	2016
	In vitro	Growth inhibition (40–200 nmol/L, 24–48H); apoptosis induction (Hep3B)[BF]	Apolipoprotein E (APOE) knockdown induced Na^+^/K^+^-ATPase, caveolin, PI3K/AKT/GSK3b and apoptosis signal cascades↑;Cyclin D1, Cdc25c, Cdc2↓	Liu et al. [166]	2016
	In vitro	Growth inhibition (0.04 μg/mL, 72H); S- and G2-phase arrest; apoptosis induction; anti-migration and invasion; adhesion inhibition (HCC-LM3) [BF]	E-cadherin↑; β-catenin, MMP-2, -9, VEGF↓	Sheng et al. [67]	2016
	In vitro	Growth inhibition (0.1 mg/mL, 24H); apoptosis induction (HepG2, HLE) [HCS]	Bcl-2↑;ΔΨm, Bax, Bid, cytochrome *c*↓	Xia et al. [167]	2017
Leukemia	In vitro	Vitamin D-induced cell differentiation enhancing (7.5, 10, 12.5 nM, 24H); VDR transactivation activity enhancing (HL-60, THP-1, U937) [BF]	1,25(OH)2D3-induced CYP24A1, CD14, CAMP, PTGS1, CD11b, CDKN1A expression, nuclear VDR expression, histone acetylation and VDR recruitment to the CYP24A1 promoter, Erk MAP kinase activation↑	Amano et al. [168]	2009
	In vitro	Proliferation inhibition (5–80 nmol/L, 12–72H); apoptosis induction (NB4) [BF]	Synergized with PD98059; caspase-3 activation↑; survivin expression↓	Zhu et al. [169]	2012
	In vitro	Reverse multidrug resistance (0.001–0.1 μM, 48H); S-phase arrest (K562, K562/VCR) [BF]	Bax↑; MRP1, Bcl-xL↓	Zhai et al. [136]	2014
	In vitro	Growth inhibition (0.01–0.5 μmol/L, 48H); apoptosis induction; cell cycle arrest (HEL) [BF]	WT1 gene methylation, DNMT3a, DNMT3b protein↑; WT1 mRNA expression↓	Wang et al. [137]	2017
Lung	In vitro	Growth inhibition (0.1 μM, 24–48H); apoptosis induction; ROS-dependent mitochondrial dysfunction (ASTC-a-1) [BF]	ROS production, Bax translocation from cytosol to mitochondria, caspase-3 activation↑	Sun et al. [50]	2011
	In vitro	Growth inhibition (2.5–10 μM, 48–72H); apoptosis induction; G1-phase arrest (A549) [BF]	Cyto C (Cytosol)/Cyto C (mitochondrial), cleaved-caspase-3, cleaved-PARP, p53, p21Waf↑; Bcl-2/Bax ratio, Cyclin D1, COX-2, p-VEGFR2, p-VEGFR1, p-EGFR, p-Akt, p-p38MAPK, p-NKκB, p-ERK1/2↓	Jiang et al. [46]	2010
	In vitro	Growth inhibition (20–100 nM, 48–72H); apoptosis induction (A549) [BF]	Bax, cleaved-caspase-3↑; Bcl-2, livin, p-Akt↓	Zhu et al. [138]	2012
	In vitro	Growth inhibition (20 nM, 72H); reverses HGF-resistance to EGFR-TKIs; apoptosis induction (HCC827, PC-9, H1975) [BF]	Cleaved-caspase-3, -9, cleaved-PARP↑; blockage of Met/PI3k/Akt pathway	Kang et al. [170]	2013
	In vitro	Growth inhibition (1–4 μM, 24H); induce DNA condensation (NCI-H460) [BF]	DNA-PK, BRCA1, 14-3-3-σ, MDC1, MGMT, P53↓	Wu et al. [70]	2014
	In vitro	Growth inhibition (2–8 ng/mL, 48H); apoptosis induction (A549) [BF]	Cleaved-caspase-3, cytosol cytochrome *c*↑; ΔΨm↓	Ding et al. [41]	2014
	In vitro	TGF-β induced epithelial-to-mesenchymal transition and migration inhibition (50 nM, 24H) (A549) [BF]	TGF-β receptor, I and II TGF-β induced E-cadherin, p-Smad2, p-Smad3↓	Zhao et al. [79]	2015
	In vitro	Invasion and migration inhibition (25–100 nM, 24–48H); cell adhesion inhibition (NCI-H460) [BF]	RhoA, MMP-2, -9 expression, TIMP1 expression↑; MMP-2, -9 activity, NF-κB, PKC, GRB2, p-AKT, p-ERK, p-P38, p-JNK1/2, ROCK1, FAK, TIMP2↓	Wu et al. [70]	2015
	In vitro	Growth inhibition (5–20 nmol/L, 72H); cellular proteasome activity inhibition; ubiquitinated proteins accumulation (A549) [BF211]	PSMB6 β1↓	Sun et al. [92]	2016
	In vitro	Growth inhibition (1–2 nM, 48H); enhanced HDAC inhibitors induced apoptosis (A549) [BF]	SRC-3p-Akt, Bcl-2↓	Zou et al. [155]	2016
	In vitro and in vivo	Growth inhibition (1–4 μM, 12–48H); cell morphological changes induction; DNA condensation; apoptosis induction (H460) [BF]	Cytochrome C, Apaf-1, active caspase-3, FasL/CD95, FasL, AIF, Endo G, caspase-9 activity, GADD153 mRNA expression, ROS production↑; pro-caspase-3, Bcl-2, GRP78 mRNA expression, ΔΨm↓	Wu et al. [150]	2017
	In vitro and in vivo	Growth inhibition (12.5–50 nM, 1–12H); apoptosis induction (A549) [BF]	Cleaved-caspase-3, cleaved-PARP, Caspase-3 activity↑; p-Src↓	Liu et al. [36]	2016
	In vitro	Inhibits gefitinib resistant cell migration and invasion (2.5–10 nM, 24–48H) (NCI-H460) [BF]	p-p38, p65↑; SOS-1, MMP-2, RhoA, N-Cadherin, E-Cadherin↓	Huang et al. [74]	2016
	In vitro	Growth inhibition (25–100 nM, 6–48H); apoptosis induction (H1975) [BF]	Mcl-1 protein degradation, cleaved-PARP, cleaved-caspase-3, Bax, Bak↑; p-GSK-3β, Bcl-1, Bcl-2, Bcl-xL↓	Kang et al. [171]	2017
	In vitro	Growth inhibition (1–100nmoL/L, 24–72H); apoptosis induction; S-phase arrest (A549) [BF]	Caspase-3↑	Zhang et al. [172]	2017
Melanoma	In vitro	Growth inhibition (150–550 nM, 24–48H); apoptosis induction (A375.S2) [BF]	ROS production, intracellular Ca^2+^ production, NO formation; cleaved-caspase-3, -8, -9, cytochrome *c*, AIF, Endo G, Bax, Fas, FasL, GRP78; ΔΨm, Bcl-xL↓	Hsiao et al. [44]	2012
Myeloma	In vitro	Growth inhibition (20 nM, 48H); apoptosis induction; G2/M arrest (U929, U266) [BF]	Chemosensitivity↑; PARP1↓	Huang et al. [173]	2013
	In vitro and in vivo	Growth inhibition (12 nM, 12–48H); synergistic with MK2206 surpassed bortezomib resistance (U929, U266) [BF]	p-Akt, p-mTOR↑; p-P70, IL-6 secretion↓	Xiang et al. [147]	2017
Oral	In vitro	Growth inhibition (125 nM, 24H); G0/G1 phase arrest; apoptosis induction (CAL27)[BF]	Cytochrome c, Apaf-1, AIF, cleaved-caspase-3, -9↑; p-Akt, Cyclin D1, p-Bad↓	Tsai et al. [139]	2012
	In vitro	Growth inhibition (50–150 nM, 24–48H); apoptosis induction (CAL27) [BF]	Intracellular ROS accumulation, p-JNK, p-p38, p-c-Jun↑; hTERT expression ↓	Tian et al. [140]	2015
Osteosarcoma	In vitro	Growth inhibition (25–50 nM, 24–48H); anti-invasion and migration (U2OS) [BF]	SOS-1, JNK1/2, ERK1/2, p-38, MMP-7, -9 enzyme activity↓	Chueh et al. [69]	2011
	In vitro and in vivo	Growth inhibition (25 nM, 24H); apoptosis induction; G2/M arrest (U2OS, U2OS/MTX300) [BF]	Cleaved-PARP↑; Hsp27, p-Akt, P65↓	Xie et al. [86]	2012
	In vitro	Inhibition of differentiation and proliferation (10 μM, 72Η) (hMG63-derived cancer stem cell) [BF]	CD133↓	Chang et al. [174]	2014
	In vitro	Growth inhibition (10–40 nM, 24H) (MG-63) [BF]	ROS production, mitochondrial membrane hyperpolarization, Apaf-1, cleaved-PARP, cleaved-caspase-3, -7, -9↑; Bcl-2/Bax ratio↓	Wang et al. [51]	2014
	In vitro	Inhibition of differentiation and proliferation (10 μΜ, 8 days) (Primary osteosarcoma stem cell C1OS) [BF]	Cleaved-caspase-3, miR-148a↑; ALDH1, hTERT, Nanog. CD133, Notch, Bmi-1↓	Chang et al. [175]	2015
	In vitro	Growth inhibition (10–50 μg/L, 6–24H); apoptosis induction (U-2OS) [BF]	Cytosol/mitochondrial cytochrome c, cleaved-PARP, cleaved-caspase-3, -9, Bax↑; ΔΨm, PARP, Bcl-2↓	Chen et al. [40]	2016
	In vitro	Growth inhibition (0.05–10 μM, 24H); apoptosis induction (U-2OS, Saos-2) [BF]	ROS production, BBC3↑; miR221↓	Zhang et al. [141]	2016
	In vitro	Growth inhibition (200 nM, 6–48H); apoptosis induction (U-2OS) [BF]	Ca^2+^ release, caspase-3, -8, -9 activity, cytochrome *c*, Fas-L, cleaved-PARP, Calpain 1, ATF-6α, GRP-78, caspase-4↑; ΔΨm, Bcl-2, Bcl-xL↓	Lee et al. [176]	2017
Ovarian	In vitro	Growth inhibition (1 ng/mL, 48H); G0/G1 arrest; apoptosis induction [BF] (SK-OV-3, OMC-3)	p21, cleaved-caspase-9↑; Cyclin A, Cyclin D3, Bcl-2, Bcl-xL↓	Takai et al. [132]	2008
	In vitro	Growth inhibition (1–100 ng/mL, 48H); apoptosis induction (SKOV3, ES-2) [BF]	miR-183 downregulation enhanced bufalin-induced growth inhibition and apoptosis	Chen et al. [177]	2016
Pancreatic	In vitro and in vivo	Growth inhibition (0.001–0.1 μM, 48H); apoptosis induction; enhance sensitivity to gemcitabine (Bx-PC3, MiaPaCa2, Panc-1) [BF]	ASK1, p-JNK, cleaved-caspase-3↑; Ki-67, Bcl-2↓	Chen et al. [148]	2012
	In vitro	Growth inhibition (50–200 nM,48H); G2/M arrest; apoptosis induction; enhanced gemcitabine chemosensitivity (Panc-1, CFPAC-1) [BF]	Bax, P21↑; Bcl-2, pro-caspase-3, -9, CyclinB1, CDK1↓	Li et al. [47]	2014
	In vitro	Growth inhibition (50–150 nM, 24–48H); mitochondria-dependent apoptosis induction; (Capan-2)[BF]	p-JNK, p-p38, p-c-Jun, intracellular ROS accumulation↑; hTERT expression↓	Tian et al. [140]	2015
	In vitro and in vivo	Cancer stem cells formation inhibition (50 nM, 24H) (MiaPaCa2/GEM) [BF]	CD24 expression, ESA expression, PTCH1, PTCH2, Gli1↓	Wang et al. [82]	2016
	In vitro and in vivo	Growth inhibition (0.1–10 μM, 24H); S-phase arrest (SW1990, BxPc3) [BF]	c-Myc, NF-κB↓	Liu et al. [149]	2016
Prostate	In vitro	Growth inhibition (0.1–10 μM, 24H); apoptosis induction (LNCaP, PC3, DU145) [BF]	Caspase-3 activity, caspase-9 activity, intracellular Ca^2+^↑	Yeh et al. [178]	2003
	In vitro	Growth inhibition (15 μM, 24H); apoptosis induction (PC3) [BF]	miR-181a induction enhanced bufalin-induced growth inhibition and apoptosis; miR-181a expression, caspase-3 activity↑; Bcl-2↓	Zhai et al. [179].	2013
Renal	In vitro	Induce new high density glycogen-microtubule structures formation (1–20 nM, 0–4.5H) (ACHN) [BF]	K^−^ATPase-induced ERK1/2 phosphorylation↑	Fridman E et al. [180]	2012
Tongue	In vitro	Growth inhibition (100–500 nM, 48H); G2/M arrest; mitochondria-dependent apoptosis (SCC-4) [BF]	Ca^2+^, NO production, DR5 expression, caspase-9 expression↑; Bcl-2, Bid, calpain 1, ATF-6β, ΔΨm, ROS production↓	Chou et al. [143]	2017

### Induce cell death other than apoptosis

In contrast to the apoptosis-inducing activity of Bufalin, well-documented in various cancer cell lines, 100 nM of Bufalin does not induce caspase-dependent apoptosis in human colorectal caco-2 and HT-22 cell lines [59]. Instead, Bufalin induces cell death by triggering autophagy, possibly through a ROS- and JNK-dependent pathways in colorectal cancer cells, followed by increased expression of ATG5 and Beclin-1 without significant induction of apoptosis, PARP cleavage, and caspase-3 cleavage [59]. It may be plausible that Bufalin enhances the radiosensitivity of colorectal cancer through ROS-mediated autophagy, which deserves further investigation [59].

In addition to inducing apoptosis-dependent cell death, laboratory studies have suggested that Bufalin (0.1–1 μM) inhibits Na^+^/K^+^-ATPase activity, a crucial ion pump, and transducer ligand receptor. It suppresses cancer cell proliferation and possesses synergistic effect with Sorafenib against liver cancer [60, 61]. Changes in Na^+^/K^+^-ATPase activity play an important role in cell survival and function, and high expression of Na^+^/K^+^-ATPase subunit α is associated with poor overall survival in liver cancer patients [27]. Moreover, compared with adjacent tissues, the expression of Na^+^/K^+^-ATPase subunit α1 was significantly elevated in liver cancer tissues, suggesting that the specific targeting of Na^+^/K^+^-ATPase can have therapeutic effects on cancer cells without noticeable effect on normal cells [27]. The role of Na^+^/K^+^-ATPase in mediating cytotoxicity of Bufalin should be highlighted, and further studies are warranted.

### Metastasis inhibition (angiogenesis, MMPs, EMT, others)

#### Angiogenesis

Hyperactive angiogenesis is a hallmark of cancer cells. It not only provides oxygen and extra nutrient, maintains the high proliferation rate of cancer cells, but also promotes local and distant metastasis of malignant cells [62]. Bufalin had been reported to inhibit cancer cell migration and invasion in liver cancer and lung cancer cell lines by down-regulating vascular endothelial growth factor (VEGF), which plays a major role in tumor angiogenesis [46, 63, 64]. Anti-angiogenic drugs, such as Sorafenib, may be complicated by relatively easy-acquired drug resistance, rapid onset of relapse after discontinuation, and the potential for tumor metastasis. A previous study revealed that Bufalin enhances the anti-angiogenic effect of Sorafenib via AKT/VEGF signaling [64]. The co-administration of Bufalin and Sorafenib provides a novel therapeutic option for patients with advanced HCC and is worthy for further in-depth investigations [63, 64].

#### MMPs

Matrix metalloproteinases (MMPs) are a class of enzymes that degrade extracellular matrix proteins and are thought to play important roles in cell proliferation, migration, angiogenesis, and etc. In cancer cells, especially those with high metastatic potentials, MMPs, in particular, MMP-2 and MMP-9, are highly expressed [65]. MMP-2 and MMP-9 primarily control cancer cell motility, and down-regulation of these proteins was reported across studies to reduce cancer metastasis [66, 67]. Bufalin was found to down-regulate MMPs in human liver cancer, lung cancer, osteosarcoma, and etc. [68–70]. In human HCC, Bufalin inhibits metastasis both in vitro and in vivo through pathways including the Akt/GSK3β signaling, E-cadherin/β-catenin nuclear translocation [66, 71–73]. In lung cancer cells, Bufalin (25–10 nM) was observed to suppress MMP-2 and MMP-9 expression, which were mediated through multiple pathways, including p38 MAPK, c-Jun N-terminal kinase (JNK), extracellular signal-regulated kinase1/2 (Erk1/2), focal adhesion kinase (FAK), Rho-associated protein kinase 1 (ROCK1) and NF-κB [69, 70, 74]. Bufalin is a broad inhibitor of various inflammatory signaling pathways in cancer. It was noticed that in human hepatoma cells, the expression of the inflammatory protein COX-2 is down-regulated and is associated with the inhibition of cell invasion and migration [66].

#### EMT

The epithelial–mesenchymal transition (EMT) is a process in which epithelial cells malignant phenotypes to become mesenchymal stem cells that promote metastasis. Bufalin was found to inhibit EMT in cervical, colorectal, hepatic, lung cancer by downregulating mesenchymal markers and upregulating epithelial markers [66, 67, 71, 75–80]. Bufalin can attenuate transforming growth factor-β (TGF-β)-induced upregulation of N-cadherin, vimentin, and Snail while downregulating E-cadherin by targeting hypoxia-inducible factor-1α (HIF-1α) [76]. In addition, the regulating of motility and invasiveness of cancer cells may be related to the PI3K/AKT/mTOR pathway [76, 81], Wnt/β-catenin pathway [66, 77], Hedgehog signaling pathway [67, 82], and etc.

#### Others

Bufalin is reported to act as a natural anti-inflammatory small molecule that upregulates osteoprotegerin and down-regulates receptor activator of NF-κB ligand (RANKL), ameliorating cannabinoid 2 receptor (CB2)-mediated cancer-induced pain and bone destruction [83]. Bufalin also down-regulated several metastasis-related genes, such as Rho-associated (Rho A), integrins and FAK [70, 80, 84, 85]. A recent study revealed that bufalin combined with sorafenib synergistically inhibited liver cancer cell migration by targeting mTOR/Akt/VEGF signaling and affecting tumor vascular microenvironment [63, 64].

### Omics approach in the study of antineoplastic effects of Bufalin and Huachansu

Few genomics and proteomics approaches are used to study the anti-cancer effects of Bufalin or HCS. Using a comparative proteomics approach, Xie et al. [86, 87] identified that Bufalin modifies 24 differentially expressed protein, particularly, the expression of proteins involve in cell metabolism, apoptosis, and cytoskeleton structure. Among them, the heat shock protein 27 (Hsp27) decreased remarkably, and its down-regulation played a critical role in bufalin-induced apoptosis in osteosarcoma cells [86, 87]. Another study used two quantitative proteomics methods, isobaric tags for relative and absolute quantification (iTRAQ)-based and label-free proteomic analysis, to study the target-related proteins of Bufalin in human A549 lung cancer cells [88]. The number of the differentially expressed protein commonly found in the two methods is 45 proteins, suggesting that the involvement of oxidative stress and the fibronectin-related pathways are important pathways for the anti-cancer effect of Bufalin [88]. Wu et al. [89] reported in 2014 that Bufalin modulates about 165-apoptosis-related genes in human lung cancer CNI-H460 cells using Affymetrix GeneChip. These results provide a deeper understanding of the anti-proliferative and cytotoxic mechanisms of Bufalin at the genetic level in gene assays [89]. Although a small number of proteomics studies and gene chip arrays have studied the target-related proteins or genes involved in the anti-cancer effect of Bufalin, which can serve as a paradigm for further studies of the molecular basis of Bufalin against various types of tumors, there is a lack of transcriptome analysis and other high-throughput screening in examining or predicting cell response to Bufalin treatment at both cytotoxic level and non-cytotoxic level. There is a need for high-throughput analysis to better associate the change of mRNA levels correlated with protein–protein interactions or protein–DNA interactions [90]. Further work is warranted on the molecular mechanism of Bufalin and HCS against various tumors.

## Drug delivery and its derivatives

Bufalin participates in complex cell-signal transduction pathways, which contribute to its suppressive effect of tumor progression in various cancer types. However, its structural similarity to digitoxin, digoxin and other cardiac glycosides accounts for the toxic effects at a high dosage. Previous studies suggest that Na^+^/K^+^-ATPase is a potential drug target that contributes to the emerging role of cardiac glycosides in selectively controlling tumor proliferation, but does not affect normal cell growth [36]. In the recent years, emerging newly discovered HCS derivatives have been shown to process anti-tumor effects, such as BF211, have been shown to inhibit colorectal, gastric, and lung cancer [91, 92].

In the process of seeking to improve the anti-cancer properties of Bufalin, several drug carriers have been synthesized with Bufalin, such as carbon-based nanomaterials, including liposomes and polymeric microspheres, folate receptor-targeted supramolecules, or [18F]fluoroethyl conjugates [93–101]. Structurally modified and synthetic Bufalin-loaded nanoparticles are designed to promote tumor-specific drug release and cytotoxicity, increase cellular uptake, and improve bio-distribution at the tumor site [99, 102, 103]. Some studies have reported that internalization of the Bufalin nanoparticles may at least partially contribute to the increased intracellular uptake of Bufalin in cancer cells [85, 93, 95, 102, 104–106]. However, there is still a need for better therapeutic materials with good drug solubility, well binding affinity, high tumor-specific targeting, low systemic toxicity and rapid clearance.

## Potential cardiotoxic property of Bufalin and the side-effects of toad extract and HCS in clinical studies

As a cardioactive steroid, Bufalin has a variety of biological activities, such as cardiotonic, blood pressure stimulation, and etc. At high dosages, cardioactive steroids can cause cardiac arrhythmias and exhibit cardiotoxicity. The concentration of CS (400 ng/mL) extract and Bufalin alone can induce myocardial cell arrest within a few seconds after administration by altering intracellular calcium storage in cardiomyocytes and possibly acts on sites other than the Na^+^/K^+^-ATPase [107]. Koh et al. [108] reported that zebrafish larvae responded to 100 µM Bufalin and showed a decrease in heart rates, early depolarization and polymorphic arrhythmia-like changes. Another study found that the addition of Bufalin to guinea pig papillary muscle (0.4 µmol/L) or atrium (225 nmol/L) preparations can lead to arrhythmias [109]. Further study on the effect of ethanol extract of Chinese toad venom (EET) on mice showed that 5 mg/kg of EET caused liver toxicity cardiomyocytes injuries [110–112]. In a Pilot study of HCS in patients with HCC, non-small cell lung cancer, or pancreatic cancer showed that side effects that may have been related to HCS were hematologic (thrombocytopenia and leukopenia), gastrointestinal (loss of appetite, constipation and diarrhea), mucocutaneous (dental ulcers and rashes), and cardiovascular (premature ventricular contraction and hypertension) in nature. Others include myalgia, dyspnea and dizziness [12]. However, when using HCS, no dose-limiting toxicity (DLT) was observed at doses up to 8-times higher than the usual dose used in China (conventional doses = 20–25 mL; highest dose = 162 mL). To the best of our knowledge, the typical therapeutic doses of HCS have not found significant side-effects in clinical studies. However, it has been reported that HCS can reduce the side-effects of chemotherapy and radiotherapy in gastric cancer, lung cancer, colorectal cancer, and etc. [9, 11, 113–116]. Due to the potential toxic effects of other cardiac glycosides, careful clinical evaluation should be performed prior to Bufalin administration.

## Clinical trials

Huachansu injection is a sterile hot-aqueous extract of dried toad skin, is approved by the Chinese FDA for use at oncology clinics in China. Since the 1970s, various clinical studies in China have demonstrated the anti-cancer properties of HCS, with a total response rate of 10% and 16% for patients with advanced liver and lung cancer, respectively. In a previous clinical study, the quality and consistency of three separate lots of HCS were evaluated [12]. It was found that and levels of bufalin and resibufogenin in HCS were 18.0–19.5 ng/mL, and 17.7–19.0 ng/mL, respectively [12]. The variation between three lots of HCS was remarkably close, with less than 10% variation. In the detection of bufadienolides concentration after i.v. infusion of HCS in human plasma specimens, the levels of Bufalin, cinobufagin, cinobufotalin, and resibufogenin in the used in a phase I clinical trial was 14.3 ± 0.03, 3.35 ± 0.1, 21.5 ± 0.22, and 24.5 ± 2.18 ng/mL, respectively [12]. As for Bufalin, which studies have shown is the major bufadienolide with the most pronounced anti-cancer activity, was further evaluated its concentration in plasma by liquid chromatography with mass spectrometry (LC/MS/MS) and found a maximal plasma levels at the end of the 2-h infusion which is proportional to the amount of drug administered (0.81–3.38 ng/mL) [12]. In a clinical phase I study of HCS in patients with HCC, non-small cell lung cancer, or pancreatic cancer published in 2009 and found that HCS is partially effective on cancer patients [12]. Only mild adverse events were observed, with doses up to five times the conventional clinical dose, which is approximately 15 mL of drug per meter squared of body mass (mL/m^2^) [12]. Furthermore, a phase II randomized controlled trial (RCT) of patients with advanced pancreatic adenocarcinoma (PaCa) treated with HCS in 2012 reported no clinical benefit with the addition of HCS to gemcitabine, which is similar to over 30 previously published well-designed RCTs in patients with advanced PaCa evaluating gemcitabine in combination with other cytotoxic or biologic agents [117]. However, the lack of efficacy observed in these trials does not preclude the possible efficacy of HCS in other solid malignancies. There is an increase in related publications later (Fig. 1a), indicating an increasing research interest on the potential use of HCS and its derived steroidal cardiac glycosides in cancer therapy. However, the report on the outcome of any RCT published after the phase II clinical trial is not yet available.

## Discussion and future perspectives

At present, TCM is practiced all over the world. Natural products have long been an important source of cancer treatment. CS has been used for thousands of years in the practice of TCM in aqueous extract form for the treatment of cancer. HCS has been used either alone or in combination with chemotherapeutic agents. Since the phase I study of HCS in patients with liver, lung, or pancreatic cancer in 2009, there is an increase in related publications, indicating a great interest is brought on the potential use of HCS and Bufalin in human. HCS and Bufalin exhibit a wide range of biological effects in cancer, including inhibition of cell proliferation, induction of cell apoptosis, disruption of the cell cycle, inhibition of metastasis, reversing multi-drug resistance to chemotherapeutic agents, and regulation of the immune response. HCS and Bufalin also have multiple other effects such as respiratory excitation, anti-inflammation and analgesics [83, 118].

Cancer is characterized by inflammation, and the chronic inflammatory microenvironment is often associated with malignant progression. The anti-inflammatory effects of Bufalin may at least in part contribute to its anti-cancer effect targeting the tumor microenvironment. The involvement of various inflammatory mediators such as COX-2 and PGE_2_ may be produced not only by cancer cells, but also by the surrounding immune cells, fibroblasts, and endothelial cells. The production of inflammatory mediators may create a positive feedback loop to further promote cancer proliferation, angiogenesis, invasion, and metastasis. Although most studies reported the anti-inflammatory effect of Bufalin, in breast cancer, Bufalin was reported to promote inflammatory response, accompanied by increased COX-2/IL-8 expression and enhanced tumor growth in vivo [38]. The role of Bufalin on NF-κB signaling and pro-inflammatory mediators, such as COX-2, inducible nitric oxide synthase (iNOS), tumor necrosis factor alpha (TNF-α), interleukin 1 beta (IL-1β), and interleukin 6 (IL-6) are studied across diseases, including rheumatoid arthritis [119, 120], asthma [118], bacterial infection [121], and chronic inflammatory disease [122]. It is worth highlighting that Bufalin may also possess chemo-preventive potential in skin cancer when administered topically in a rodent model [123]. Future research may extend to other types of human diseases. Research on the interaction between stromal cells and tumor cells may not only broaden the potential use of Bufalin and HCS, but also helps to understand the tumor immune microenvironment, thereby providing long-lasting repression against cancer growth for future cancer treatment.

Previous studies found that the expression of Na^+^/K^+^-ATPase subunit α1 is prognostic in liver cancer patients and is significantly elevated in cancer tissue compared with adjacent tissues [27, 60]. Recent studies of cardiac glycosides suggest Bufalin may potentially represent a promising form of targeted cancer chemotherapy on Na^+^/K^+^-ATPase subunit α1 that can be safely used for long periods without severe side effects [27, 60]. Furthermore, the results indicated that the down-regulation of Na^+^/K^+^-ATPase α1 can inhibit cell proliferation, migration, and invasion in vitro, and inhibit tumorigenesis in vivo [26, 27, 124]. These increase the likelihood of selecting patients with high Na^+^/K^+^-ATPase subunit α1, may improve the therapeutic efficacy of Bufalin and HCS. It is worth noting that since June 2015, another Na^+^/K^+^-ATPase inhibitor, RX108, is structurally similar to Bufalin and is undergoing phase I clinical trials to evaluate the safety, tolerability, pharmacokinetics, and pharmacodynamic properties in patients with locally advanced or metastatic solid tumors in Australia. However, as of July 2018, there were no recent reports for phase-I development. This study, if successfully completed, may provide valuable information about the safety of targeting Na^+^/K^+^-ATPase.

In the process of identifying active anti-cancer ingredients in Traditional Chinese Medicine, some encouraging results have been achieved and we deepened our understanding of the underlying mechanism of malignant behavior. With the growing awareness of the tumor microenvironment and the wholistic immunomodulation, it is possible that agents with systemic effects may be a double-edged sword. In the identification of novel therapeutic agents, agents that target multiple signaling pathways may have a stronger anti-cancer effect, but inevitably carry more adverse effects, such as gastrointestinal burden and hematological toxicities. Studies combining conventional cancer treatments (such as chemotherapy and radiotherapy) with TCM or derived components should be interpreted with caution, as drug interactions caused by combination therapies may have a negative impact on conventional cancer treatments, reducing their effectiveness. It is also essential to further study the underlying pathophysiological mechanisms of anti-cancer effects and adverse reactions. Inevitably, there is a great need to discover long-term effects, drug use sequences, drug–drug interactions, and individual choices further for different drugs, aimed at improving patients’ quality of life and prolonging survival.

## Concluding remarks

Vigorous studies and discoveries have revealed the anti-tumor properties of Bufalin and HCS as potentially multi-targeted agents for cancer treatment. The results indicated that Bufalin inhibits tumor progression by inhibiting cancer cell proliferation via both apoptosis dependent or independent pathways, as well as inhibiting metastasis via repressing cell motility and angiogenesis. Many animal studies and clinical studies conducted over the past decade have revealed their clinical potentials, however, in the current study, long-term toxicological studies on different individuals, pharmacodynamics studies on various dosages and administration routes have been relatively lacking. Further research is needed to elucidate potential drug-drug interactions and multi-target interactions of Bufalin and HCS. Large-scale clinical trials are warranted to translate the knowledge for anti-cancer effects of Bufalin and HCS into clinical applications as effective and safe treatment options for future cancer patients. Overall, this review highlights the advances in the study of Bufalin and HCS as emerging anticancer agents over the past decade and may shed light on the future direction of Bufalin and HCS as novel anticancer agents for clinical applications.

## Highlights

- Huachansu and Bufalin possess anti-cancer effects both in vitro and in vivo.
- The multi-target and multi-pathway pharmacological actions are promising.
- Potential drug–drug interactions and multi-target interaction lacks studies.
- Further large-scale clinical trials are warranted.

## Abbreviations

TCM: Traditional Chinese Medicine
CS: Chansu
HCS: Huachansu
NF-B: nuclear Factor-κB
PGE_2_: prostaglandin E_2_
Na^+^, K^+^-ATPase: Na^+^/K^+^ pump or sodium- and potassium-activated adenosine 5′-triphosphatase
HPLC: high-pressure liquid chromatography
IC50: the 50% inhibition dose
ER: estrogen receptor
i.p.: intraperitoneal
i.v.: intravenous
COX-2: cyclooxygenase-2
IL-8: interleukin-8
MAPK: mitogen-activated protein kinase
HCC: hepatocellular carcinoma
ROS: reactive oxygen species
SRC: steroid receptor coactivator
TRAIL: tumor necrosis factor related apoptosis-inducing ligand
JNK: c-Jun N-terminal kinase
ATG5: autophagy-related gene 5
PARP: poly-(ADP-ribose) polymerase
MMPs: matrix metalloproteinases
EMT: epithelial–mesenchymal transition
VEGF: vascular endothelial growth factor
AKT: protein kinase B
GSK3β: glycogen synthase kinase 3 beta
Erk1/2: extracellular signal-regulated kinase1/2
FAK: focal adhesion kinase
ROCK1: Rho-associated protein kinase 1
TGF-β: transforming growth factor-β
HIF-1α: hypoxia-inducible factor-1α
PI3K: phosphoinositide 3-kinase
mTOR: mammalian target of rapamycin
RANKL: receptor activator of nuclear factor-κ B ligand
CB2: cannabinoid 2 receptor
Rho A: Rho-associated
Hsp27: heat shock protein 27
iTRAQ: isobaric tags for relative and absolute quantification
EET: ethanol extract of Chinese toad venom
DLT: dose-limiting toxicity
LC/MS: liquid chromatography with mass spectrometry
RCT: randomized controlled trial
iNOS: inducible nitric oxide synthase
TNF-α: tumor necrosis factor alpha
IL-6: interleukin 6
PaCa: pancreatic adenocarcinoma
RCT: randomized controlled trial
